# Fibroblast growth factor 2 (FGF2) modulates the excitability of brain noradrenergic and serotonergic neurons: possible involvement of FGFR1 and FGFR4 receptors

**DOI:** 10.3389/fphar.2026.1875046

**Published:** 2026-07-10

**Authors:** Ruslan Paliokha, Matej Racicky, Daniil Grinchii, Talah Khoury, Segev Barak, Eliyahu Dremencov

**Affiliations:** 1 Institute of Molecular Physiology and Genetics, Centre of Biosciences, Slovak Academy of Sciences, Bratislava, Slovakia; 2 Institute of Experimental Endocrinology, Biomedical Research Center, Slovak Academy of Sciences, Bratislava, Slovakia; 3 School of Psychological Sciences and the Sagol School of Neuroscience, Tel Aviv University, Tel Aviv, Israel

**Keywords:** dorsal raphe nucleus (DRN), FGF receptors 1,2, and 4 (FGFR1, FGFR2, FGFR4), fibroblast growth factor 2 (FGF2), *in vivo* electrophysiology, locus coeruleus (LC)

## Abstract

**Background:**

Fibroblast growth factor 2 (FGF2), in addition to its primary function in the connective tissue, plays also a role in the central nervous system (CNS). Thus, anxiolytic, antidepressant, and pro-addictive properties of this molecule have been reported. Our previous study showed that pro-alcohol consumption effect of FGF2 is mediated, at least in part, via its interaction with the central dopaminergic system. The present study aimed to test the hypothesis that pro-addictive and/or antidepressant-like effects of FGF2 can also be linked with FGF2-noradrenaline and FGF2-serotonin (5-HT) interactions.

**Methods:**

Adult male Wistar rats, weighing 250–350 g, were treated with the recombinant FGF2, selective inhibitors of FGFR1 (PD173074), FGFR2 (lirafugratinib), FGFR4 (BLU9931), or corresponding vehicle. The excitability of the noradrenergic neurons of the locus coeruleus (LC) and serotonergic (5-HT) neurons of the dorsal raphe nucleus (DRN) was assessed using the single-unit *in vivo* electrophysiology under chloral hydrate anesthesia.

**Results:**

We found that FGF2 stimulated the burst firing of noradrenergic neurons and inhibited the burst firing of 5-HT neurons. PD173074 decreased the density of the spontaneously active noradrenergic neurons in the locus coeruleus, whereas BLU9931 had a stimulatory effect on the burst activity of 5-HT neurons.

**Conclusion:**

The psychoactive effects of the FGF2 might be mediated, at least in part, via its interaction with the central monoaminergic circuits. The FGF2-catecholaminergic interactions are putatively mediated via FGFR1, and FGF2-5-HT-crosstalk-via FGFR4. These two receptors can be thus potential targets for the future CNS drugs.

## 1 Introduction

Fibroblast growth factor 2 (FGF2), also known as basic fibroblast growth factor (bFGF) or fibroblast growth factor beta, FGF-β, is a growth factor and signaling protein belonging to the family of the fibroblast growth factors. The primary biological function of FGF2 is to regulate the proliferation and activation of fibroblasts. As such, this biomolecule is involved in embryonic development, cell growth, morphogenesis, tissue repair, as well as tumor growth and invasion (Niederegger et al., 2026). Four types of FGF receptors (FGFR1-FGFR4) have been identified; FGF2 primarily interacts with three of them (FGFR1, FGFR2, and FGFR4) (Chellaiah et al., 1999; Helsten et al., 2015). Activation of these receptors leads to intracellular calcium signaling and protein kinase C activation and involves major signaling cascades such as PI3K/AKT, JAK/STAT and ERK pathways (Chen, 2025; Even-Chen and Barak, 2019).

Several lines of evidence indicate that FGF2, in addition to its primary function in the connective tissues, plays an important role in the central nervous system. It was reported that FGF2 reversed depressive-like behavior (anhedonia) and reduced hippocampal neurogenesis, induced by chronic unpredictable stress (Elsayed et al., 2012). In addition to the antidepressant-like, FGF2 might have a pro-addictive effect. It was reported in our previous studies that this growth factor increased the voluntary alcohol intake in rats (Grinchii et al., 2023; Even-Chen et al., 2017; Liran et al., 2020; Dremencov et al., 2021; Herburg et al., 2023; Hose et al., 2024; Even-Chen et al., 2022). Interestingly, an antagonist of the FGF2 receptor 1 (FGFR1), PD173074, had an opposite effect on alcohol consumption, suggesting that the pro-hedonic effect of FGF2 is mediated, at least in part, via FGFR1 receptors.

In our previous study (Grinchii et al., 2023) we demonstrated that the pro-addictive effect of the FGF2-FGFR1 complex is associated with its interaction with the central dopaminergic system. Specifically, we showed that FGF2 stimulated nigrostriatal dopaminergic neurons of the substantia nigra (NG) and mesocorticolimbic dopaminergic neurons of the ventral tegmental area (VTA). PD173074 had an opposite, inhibitory effect on dopaminergic neurons, that was dose dependent.

It is well established that dopaminergic, as well as serotonergic (5-HT) and noradrenergic systems of the brain are fundamental in pathophysiology and treatment of depression and addiction (Stahl, 2008). Noradrenergic and serotonergic (5-HT) circuits, in addition to dopaminergic ones, play also a role in alcohol use disorder (Elvig et al., 2021). It is thus possible that pro-addictive and antidepressant-like effects of FGF2 are mediated not only via dopaminergic, but also via noradrenergic and 5-HT pathways. To test this hypothesis, the present study aimed to examine the effect of recombinant FGF2, as well as the non-peptide ligands of its receptors, on the excitability of noradrenergic neurons of the locus coeruleus (LC) and 5-HT neurons of the dorsal raphe nucleus (DRN).

The noradrenergic neurons of the LC (Aghajanian and VanderMaelen, 1982), as well as 5-HT of the DRN (Hajós et al., 1995), exhibit two modes of firing activity: the single-spike tonic and burst-like phasic activity. The burst-like or phasic mode of firing of monoaminergic neurons, or generation of the cluster of spikes separated by millisecond-range inter-spike interval and followed by a second-range period of silence, is more efficient in terms of neurotransmission than the tonic mode of activity, when the same number of action potentials are generated in a single-spike mode (Cooper, 2002). To address these aspects of monoaminergic neurotransmission, we assessed the effect of FGF2 on the firing rate, as well as on the burst activity of noradrenergic and 5-HT neurons.

## 2 Methods

### 2.1 Animals

Adult Wistar rats, weighting 250–350 g, were obtained from the Institute of Toxicology and Breeding of Laboratory Animals, Centre of Experimental Medicine, Slovak Academy of Sciences (Dobrá Voda, Slovakia). The animals were housed in the Central Animal Facility of the Slovak Academy of Sciences, Centre of Experimental Medicine, Bratislava, Slovakia. Rats were kept in plastic cages (50 × 40 × 20 cm; maximum of 5 rats per cage) under standard laboratory conditions (temperature: 22 °C ± 2 °C, humidity: 55% ± 10%), with a 12 h light/12 h dark cycle (lights on at 7:00 a.m.), and had *ad libitum* access to food and water. Animals were allowed to acclimatize for at least 1 week prior to the start of the experiments. All experimental procedures were approved by the State Veterinary and Food Administration of the Slovak Republic (Permit No 732/2022 from January 25, 2022) and were conducted in accordance with Directive 2012/63/EU of the European Parliament and of the Council on the protection of animals used for scientific purpoА ses. Both male and female rats were used in the experiments assessing the effect of recombinant FGF2. Since FGF2-induced changes were prominent in males, the subsequent pharmacological experiments with FGFR antagonists were performed in male rats only.

### 2.2 Chemicals

Recombinant FGF2 was purchased from ProSpec-Tany TechnoGene Ltd. (Ness-Ziona, Israel). The selective FGFR1 antagonist PD173074 (Grinchii et al., 2023; Skap et al., 2000) was obtained from MedChemtronica AB (Sollentuna, Sweden). Selective antagonists of the receptors FGFR2 (lirafugratinib) (Schönherr et al., 2024) and FGFR4 (BLU9931) (Wang et al., 2017) were purchased from MedChemExpress (Monmouth Junction, NJ, United States). All other reagents were obtained from Merk, s. r.o. (Bratislava, Slovakia).

### 2.3 FGF2 administration

Recombinant FGF2 was dissolved (160 μg/mL) in phosphate-buffered saline (PBS) containing 0.1% bovine serum albumin (BSA) and administered intraperitoneally (i.p., 1 h prior to electrophysiological recordings) at 0.5 mL/kg, as described in our previous study. The final dose was 80 μg/kg. The dose of 80 μg/kg was selected based on our previous work in which the same systemic FGF2 dose produced robust effects on alcohol-related behavior and dopaminergic neuronal activity without apparent nonspecific behavioral or physiological toxicity.

### 2.4 Selective blockade of FGF receptors

PD173074 was dissolved in dimethyl sulfoxide (DMSO; 7.5 mg/mL) and administered at 2 mL/kg (i.p.; the final dose 15 mg/kg), as previously described (Grinchii et al., 2023). One micromolar DMSO solutions of lirafugratinib and BLU9931 were prepared and loaded in Hamilton micro-syringes (Hamilton Company, Reno, NV, United States). Rats were anesthetized with chloral hydrate (0.4 g/kg, i. p.) and placed in a stereotaxic frame (David Kopf Instruments, Tujunga, CA, United States). Body temperature was maintained at 37 °C using a heating pad (Gaymar Instruments, Orchard Park, NY, United States), and supplemental doses of chloral hydrate were administered when necessary. A burr hole (∼2 mm) was drilled above the left lateral cerebral ventricle (−0.8 to −1.0 mm posterior to bregma, ±1.4 to ±1.6 mm lateral from the midline; Paxinos and Watson, 2014) using a high-speed stereotaxic drill (Foredom Electric Company, Bethel, CT, United States). A needle of the Hamilton micro-syringe was lowered into the left lateral cerebral ventricle (3.5 mm ventral from the brain surface). Five microliters of lirafugratinib or BLU9931 were injected into the cerebral ventricle. The dose of PD173074 was selected based on our previous study, in which systemic administration of this FGFR1 antagonist effectively modulated FGF2/FGFR1-related effects on alcohol-related behavior and dopaminergic neuronal activity. Thus, the same dose was used here to allow comparison between the effects of FGFR1 blockade on dopaminergic neurons reported previously and on LC noradrenergic and DRN serotonergic neurons examined in the present study. The concentrations of lirafugratinib and BLU9931 were selected based on their reported selectivity toward FGFR2 and FGFR4, respectively, and were adapted for acute intracerebroventricular administration in order to target central FGFR signaling while minimizing systemic exposure. Since these antagonists were used to test receptor involvement rather than to establish a full dose-response relationship, the interpretation of these pharmacological experiments is limited to the selected experimental conditions. Animals remained anesthetized and secured in the stereotaxic apparatus until electrophysiological recordings commenced approximately 1 h later.

### 2.5 *In vivo* electrophysiology

In the rats pre-treated with lirafugratinib or BLU9931, a hole was drilled in the skull above the dorsal raphe nucleus (DRN; 8 mm posterior to the bregma and 0 mm lateral to midline) and locus coeruleus (LC; 8.1 mm posterior to bregma and 1.1 mm lateral to midline, Paxinos and Watson, 2014). Other rats were anesthetized with chloral hydrate and mounted in the stereotaxic frame, as previously explained, and the hole above the DRN or LC was drilled. Glass borosilicate electrodes were pulled using a DMZ Universal Puller (Zeitz-Instruments GmbH, Martinsried, Germany) and filled with 2 M NaCl solution (impedance 4–6 MΩ). The recorded signal was amplified using a DP-311 differential amplifier (AD Instruments, Dunedin, New Zealand), filtered from electrical noise using a HumBug noise eliminator (Carlsborg, WA, United States), and digitized via a 4/35 PowerLab data acquisition system (AD Instruments) connected to a personal computer (Lenovo 1010, SAR Hong Kong, China) using LabChart 7.5 software (AD Instruments, Dunedin, New Zealand). The sampling rate was set to 100 kHz.

Noradrenergic neurons were recorded from the LC at coordinates 8.0–8.3 mm posterior to bregma, 1.2–1.4 mm lateral to the midline, and 5.5–7.5 mm ventral to the brain surface (Paxinos and Watson, 2014). These neurons were identified by their regular firing rate (0.5–10.0 Hz), long-duration positive action potentials (0.8–1.2 m), and a characteristic burst response to nociceptive pinch of the contralateral hind paw (Aghajanian and VanderMaelen, 1982; Grinchii et al., 2022).

The 5-HT neurons were recorded from the DRN at coordinates 7.8–8.3 mm posterior to bregma, 0 mm lateral to the midline, and 4.5–7.0 mm ventral to the brain surface (Paxinos and Watson, 2014). These neurons were identified based on a regular firing rate of 0.5–5.0 Hz and long-duration positive action potential (0.8–1.2 ms) (Hajós et al., 1995; Paliokha et al., 2026).

### 2.6 Data analysis

Action potentials (spikes), generated by noradrenergic and 5-HT neurons, were detected using the spike sorting algorithm, with the version 6.02 of Spike2 software (Cambridge Electronic Design, Cambridge, United Kingdom). The neuronal firing rate and burst activity characteristics were calculated using the burstiDAtor software (www.github.com/nno/burstidator). The onset of a burst was signified by the occurrence of two spikes with ISI <0.08 s for noradrenergic, and ISI <0.01 s for 5-HT neurons. The termination of a burst was defined as an ISI >0.16 s for noradrenergic (Grace and Bunney, 1984; Dawe et al., 2001) and ISI >0.010s for 5-HT neurons (Hajós et al., 2007). Statistical assessments were performed using SigmaPlot 12.5 software (Systat Software Inc., Chicago, IL, United States). Analysis of variance (ANOVA) for repeated measures, followed by Bonferroni *post hoc* test, was used to determine the effects of FGF2 on the spontaneous firing activity of noradrenergic and 5-HT neurons in male and female rats. Two-tailed Student’s t-test was used to determine the effects of PD173074, lirafugratinib and BLU9931. The probability of p ≤ 0.05 was considered significant.

## 3 Results

### 3.1 Recombinant FGF2 enhanced the excitability of noradrenergic neurons

**FIGURE 1:**
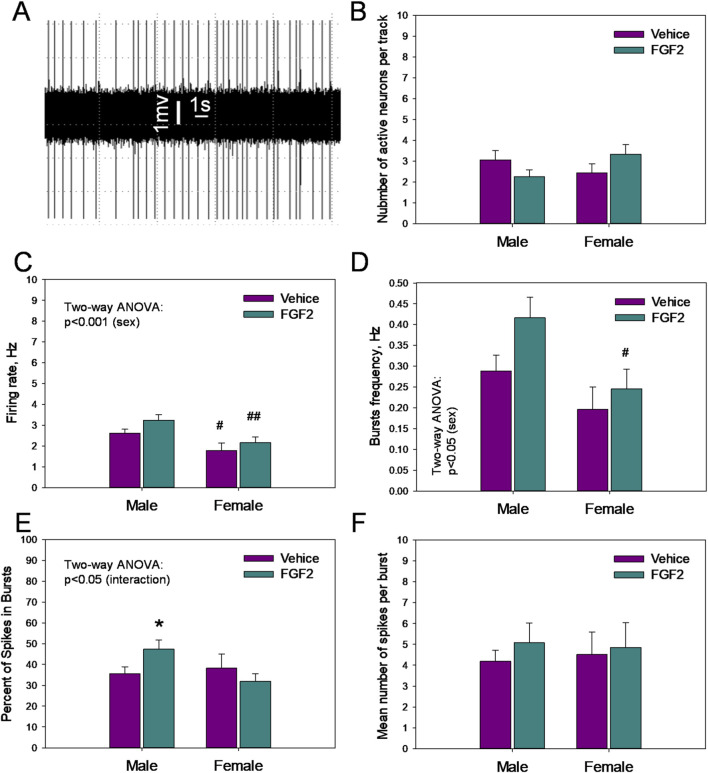
Representative recording from a LC noradrenergic neuron **(A)** and the effect of FGF2 on the **(B)** density of the spontaneously active neurons, their mean firing rate **(C)**, frequency of burst firing **(D)**, percentage of spikes occurring in bursts **(E)** and mean number of spikes per burst **(F)** in male and female rats; ^#^p < 0.05 and ^##^p < 0.01 in comparison with males and *p < 0.05 in comparison with the vehicle-treated controls, two-tailed Student’s t-test.

As FGF2 has been implicated in stress and depression, we first tested its effects on noradrenergic neuronal activity within the locus coeruleus FGF2 (80 μg/kg) was administered to the rats, and 1 h later we measured the firing activity of noradrenergic neurons (Figure 1A). The mean number of spontaneously active neurons per electrode descent did not differ between sexes or between FGF2- and vehicle-treated animals (Figure 1B; n = 51 neurons from 5 vehicle- and 38 neurons from 5 FGF2-treated males and 25 neurons from 5 vehicle- and 38 neurons from 5 FGF2-treated females).

We found that FGF2 did not affect neuronal firing rate. However, females exhibited lower firing rate compared to males, regardless of FGF2 treatment (Figure 1C; F_1,147_ = 11.33, p < 0.001, two-way ANOVA). Bonferroni *post hoc* test confirmed the sex differences for both vehicle- (p = 0.04) and FGF2-treated rats (p = 0.007). The sex × FGF2 interaction did not reach statistical significance.

Significant sex differences were also observed in burst frequency (Figure 1D; F_1,128_ = 6.11, p = 0.02; two-way ANOVA). Bonferroni *post hoc* analysis confirmed this difference in FGF2-treated animals (p = 0.02), but not in vehicle-treated rats. Again, neither the main effect of FGF2 nor the sex × FGF2 interaction was statistically significant.

A significant sex × FGF2 interaction was detected for the percentage of spikes occurring within bursts (Figure 1E; F_1,128_ = 3.98, p = 0.04; two-way ANOVA). Bonferroni *post hoc* analysis revealed an increase in this parameter in FGF2-treated males compared to vehicle-treated females (p = 0.03), as well as a significant difference between FGF2-treated males and females, with higher values in males (p = 0.01).

The mean number of spikes per burst did not differ between sexes or between FGF2- and vehicle-treated animals (Figure 1F).

Together, these results suggest that FGF2 modulates burst-related activity of LC noradrenergic neurons in a sex-dependent manner, with the effect being more prominent in male rats.

### 3.2 Antagonist of FGFR1 suppresses a population of noradrenergic neurons of the LC

**FIGURE 2:**
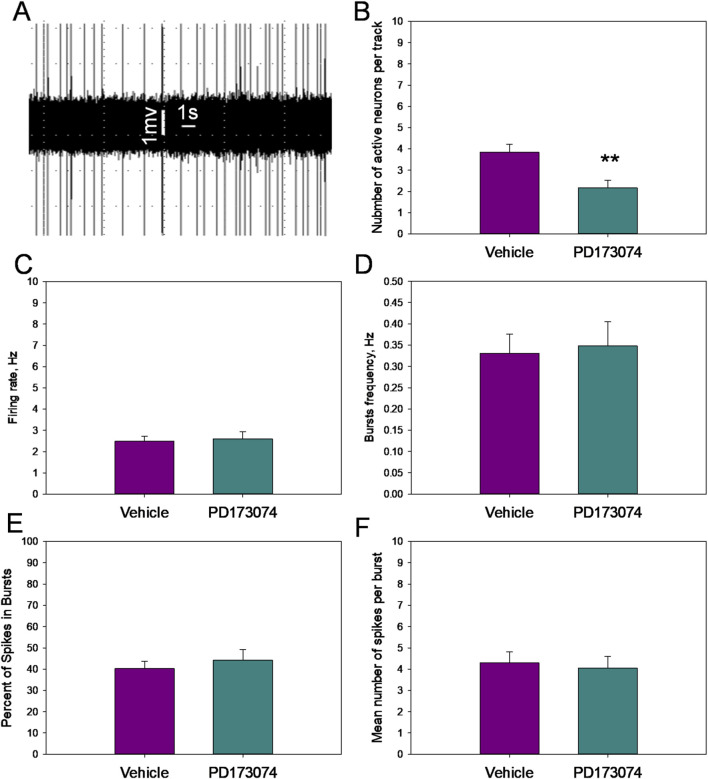
Representative recording from a LC noradrenergic neuron **(A)** and the effect of PD173074 on the **(B)** density of the spontaneously active neurons, their mean firing rate **(C)**, frequency of burst firing **(D)**, percentage of spikes occurring in bursts **(E)** and mean number of spikes per burst **(F)** in male rats; **p < 0.01 in comparison with the vehicle-treated controls, two-tailed Student’s t-test.

We injected the FGFR1 antagonist PD173074, and measured NE neuronal activity 7 h thereafter (Figure 2A). We found that PD173074 decreased the mean number of the spontaneously active neurons per electrode track (Figure 2B; p = 0.004, two-tailed Student’s t-test, n = 46 neurons from 5 vehicle-rats and 26 neurons from 5 PD173074-treated rats; B). With respect to other characteristics, such as firing rate, burst frequency, percentage of spikes occurring within the bursts and mean number of spikes per burst, it was no statistically significant effect of PD173074 (Figures 2C–F).

The decrease in active neurons per track reflects a reduction in the number of spontaneously active LC neurons encountered during electrode descents. In contrast, panels C–F describe firing properties only of neurons that remained spontaneously active and were recorded. Therefore, PD173074 may suppress the spontaneous activity of a subpopulation of LC neurons without altering firing parameters of the remaining active neurons.

Together, these results suggest that, although FGFR1 inhibition suppresses a population of noradrenergic neurons of the LC.

### 3.3 Recombinant FGF2 diminished the excitability of 5-HT neurons

**FIGURE 3:**
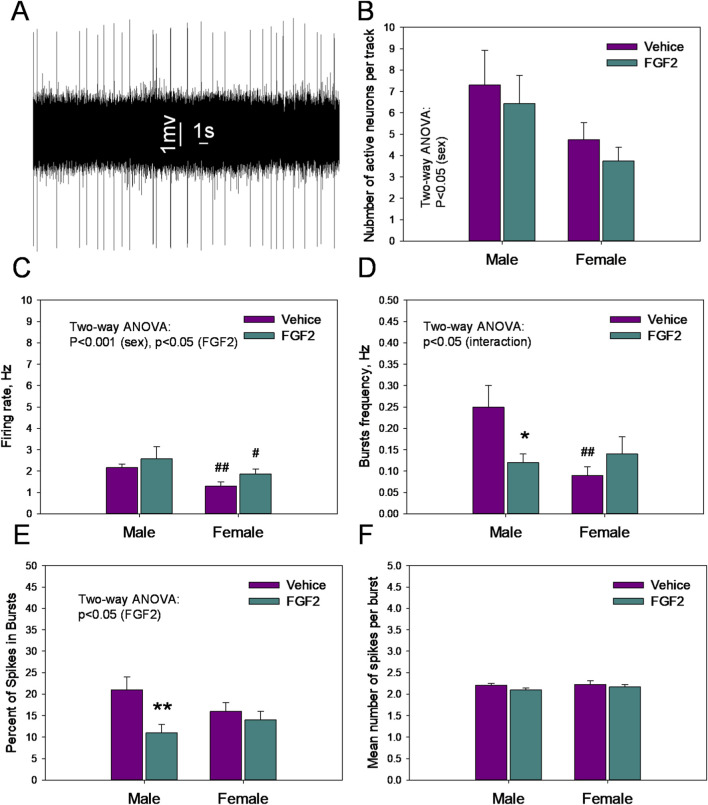
Representative recording from a DRN 5-HT neuron **(A)** and the effect of FGF2 on the **(B)** density of the spontaneously active neurons, their mean firing rate **(C)**, frequency of burst firing **(D)**, percentage of spikes occurring in bursts **(E)** and mean number of spikes per burst **(F)** in male and female rats; ^#^p < 0.05 and ^##^p < 0.01 in comparison with males and *p < 0.05 and **p < 0.001 in comparison with the vehicle-treated controls, two-tailed Student’s t-test.

As we found that FGF2-FGFR1 mechanisms affected the firing activity of noradrenergic neurons, we next tested the effects of FGF2 on serotonergic neuronal activation. First, we injected FGF2 (80 μg/kg) and recorded the firing activity of DRN 5-HT neurons (Figure 3A) 1 hour later.

It was found that FGF2 did not affect neuronal firing rate. However, females exhibited lower firing rate compared to males, regardless of FGF2 treatment (Figure 3B; F_1,36_ = 5.00, p = 0.03, two-way ANOVA, n = 78 neurons from 5 vehicle- and 45 neurons from 4 FGF2-treated males and 38 neurons from 4 vehicle- and 45 neurons from 4 FGF2-treated females). Even though Bonferroni *post hoc* test did not reveal statistical difference between any pair of the groups, the value in males tended to be higher than in females.

Similarly, significant sex differences were observed in the mean neuronal firing rate (Figure 3C; F_1,205_ = 11.40, p < 0.001). Bonferroni confirmed this difference for the vehicle- (p = 0.006), as well as for FGF2-treated rats (p = 0.047; values in males higher than in females in both cases). Even though two-way ANOVA detected a significant effect of FGF2 (F1,205 = 4.33, p = 0.04), Bonferroni *post hoc* test did not detect a difference between vehicle- and FGF2-treated animals in males or in females.

With respect to the burst frequency, it was close-to-significant effect of sex (Figure 3D; F_1,134_ = 3.77, p = 0.054) and significant sex × FGF2 interaction (F_1,134_ = 5.28, p = 0.02). Bonferroni *post hoc* test revealed sex differences in vehicle- (p = 0.003, value in males higher than in females), but not in FGF2-treated rats. Significant diminishing effect of FGF2 confirmed by Bonferroni *post hoc* test for males (p = 0.02), but not for females.

Regarding the percentage of spikes occurring in bursts, statistically significant effect of FGF2 was detected (Figure 3E; F_1,134_ = 4.54, p = 0.04). Bonferroni *post hoc* test confirmed statistically significant suppressing effect on FGF2 for males (p = 0.009), but not for females.

The mean number of spikes per burst did not differ between sexes or between FGF2- and vehicle-treated animals (Figure 3F).

Together, FGF2 decreases the excitability of the DRN 5-HT neurons.

### 3.4 Antagonist of FGFR4, but not the antagonists of FGFR1 and FGFR2, stimulate DRN 5-HT neurons

**FIGURE 4:**
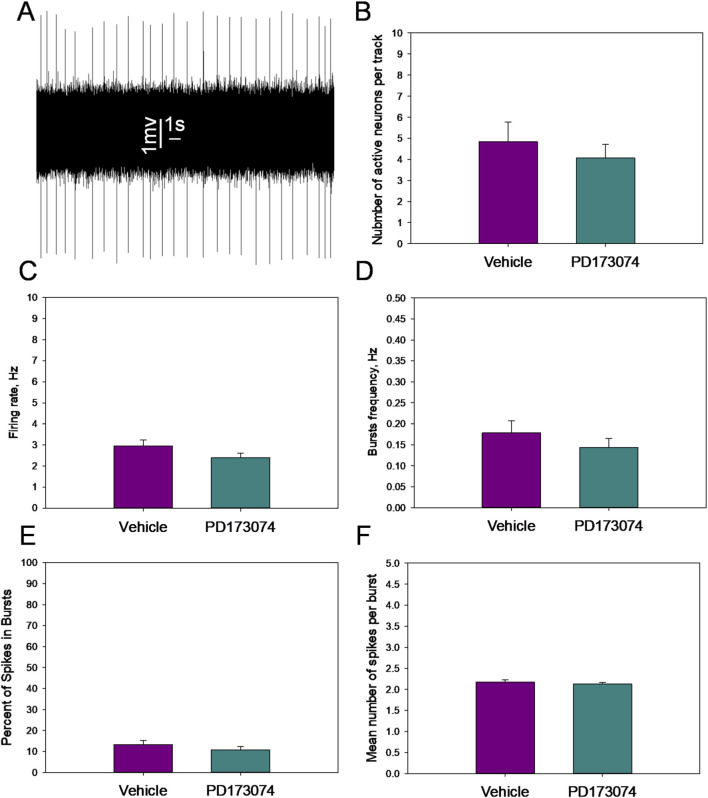
Representative recording from a DRN 5-HT neuron **(A)** and the effect of PD173074 on the **(B)** density of the spontaneously active neurons, their mean firing rate **(C)**, frequency of burst firing **(D)**, percentage of spikes occurring in bursts **(E)** and mean number of spikes per burst **(F)** in male rats.

We administered the FGFR1 antagonist PD173074 and measured 5-HT neuronal activity 7 hours thereafter (Figure 4A). No effect on the density of the spontaneously active neurons, their firing rate, or burst firing were observed (Figures 4B–F; n = 58 neurons from 5 vehicle-rats and 57 neurons from 5 PD173074-treated rats).

**FIGURE 5:**
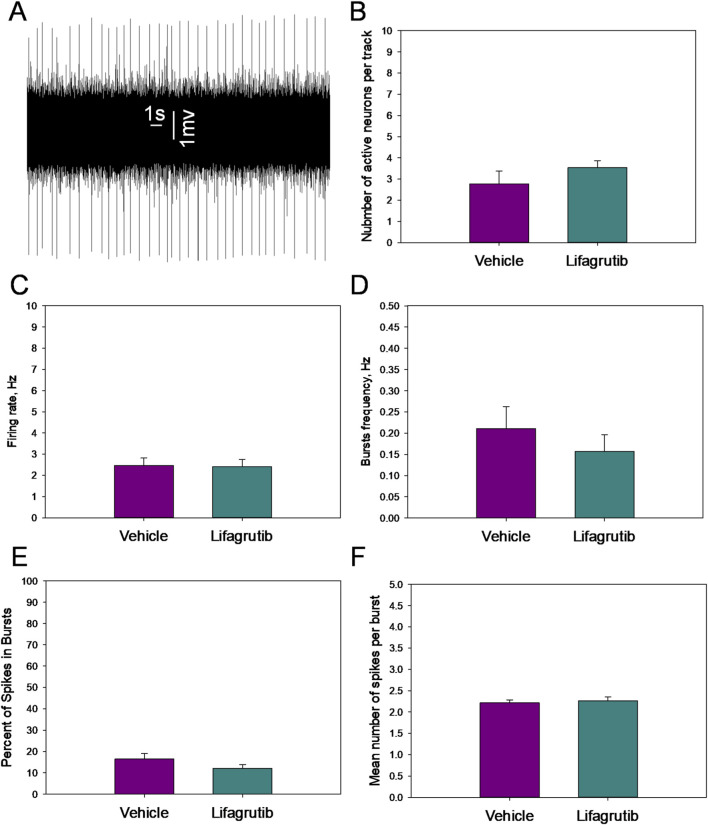
Representative recording from a DRN 5-HT neuron **(A)** and the effect of lirafugratinib on the **(B)** density of the spontaneously active neurons, their mean firing rate **(C)**, frequency of burst firing **(D)**, percentage of spikes occurring in bursts **(E)** and mean number of spikes per burst **(F)** in male rats.

The FGFR2 antagonist lirafugratinib was administered i. c.v. one hour before electrophysiological recordings (Figure 5A). No effect on the density of the spontaneously active neurons, their firing rate, or burst firing were observed (Figures 5B–F; n = 36 neurons from 5 vehicle-rats and 54 neurons from 5 PD173074-treated rats).

**FIGURE 6:**
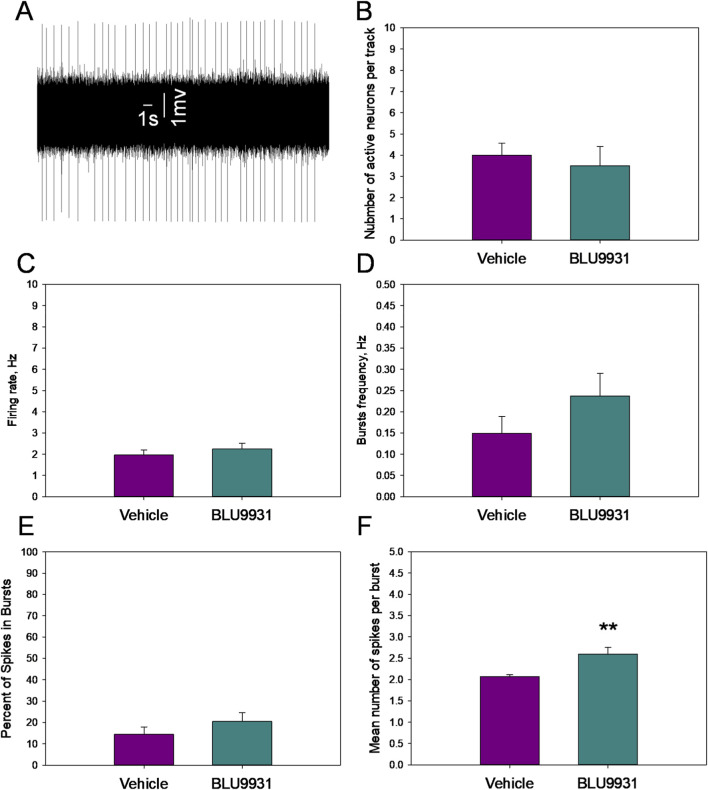
Representative recording from a DRN 5-HT neuron **(A)** and the effect of BLU9931 on the **(B)** density of the spontaneously active neurons, their mean firing rate **(C)**, frequency of burst firing **(D)**, percentage of spikes occurring in bursts **(E)** and mean number of spikes per burst **(F)** in male rats; **p < 0.01 in comparison with the vehicle-treated controls, two-tailed Student’s t-test.

The FGFR4 antagonist BLU9931 was micro-injected into the lateral brain ventriculi, and the electrophysiological recordings were performed 1 h thereafter (Figure 6A). No effect on the density of the spontaneously active neurons, their firing rate, bursts’ frequency, or percentage of spikes occurring within the bursts were observed (Figures 6B–E). BLU9931, however, significantly (Figure 6F; p = 0.004, two-tailed Student’s t-test, n = 31 neurons from 4 vehicle-rats and 48 neurons from 5 PD173074-treated rats) increased the mean number of spikes per burst.

Together, antagonist of FGFR4, but not the antagonists of FGFR1 and FGFR2, stimulate DRN 5-HT neurons.

## 4 Discussion

The results of the present study indicate that FGF2 stimulates the burst firing of noradrenergic neurons and inhibits the burst firing of 5-HT neurons in male, but not in female rats. The density of the spontaneously active noradrenergic neurons in the LC was decreased by inhibition of the FGFR1, the main receptor of FGF2, suggesting that the stimulatory effect of FGF2 on noradrenergic system might be mediated via the FGFR1. In contrast, inhibition of the FGFR4 receptor, but not of the FGFR1 or FGFR2 receptors, increased the excitability of 5-HT neurons, suggesting that this receptor putatively mediates FGF2-5-HT interactions. Together, our results suggest that FGF2 affects the noradrenergic and serotonergic systems in an opposite manner, and that its effects on the two neuromodulation systems might be linked with different FGF receptors.

With respect to noradrenergic neurons of the LC, we found that FGF2 increased the percentage of the spikes occurring in bursts. This effect was observed in male, but not in female rats. Since the burst firing of noradrenergic neurons is more efficient in terms of neurotransmitter release from the nerve terminals (Cooper, 2002), FGF2 may stimulate central noradrenergic neurotransmission. To our best knowledge, our study was first to examine the effect of the exogenous FGF2 on the excitability of the central noradrenergic neurons. It was however previously reported that FGF2 immunoreactivity exists in the brain noradrenergic neurons, suggesting FGF2 involvement in their development and/or functioning (Chadi et al., 1993). It was also reported that FGF2 regulated the expression of noradrenaline transporter (NAT) in cultivated sympathetic neurons (Sieber-Blum and Ren, 2000). It is thus possible that the stimulatory effect of FGF2 on noradrenergic neurons, observed in our study, is also explained by the interaction between FGF2 and NAT. Future studies should be performed to examine this hypothesis. The dose of FGF2 should be considered pharmacological and was selected to reproduce previously observed behavioral and dopaminergic electrophysiological effects of systemic FGF2, rather than to mimic a defined endogenous physiological concentration.

The results of our study demonstrated that PD173074 significantly decreased the density of the spontaneously active neurons of the LC. To our best knowledge, we were first to examine the effect of the FGFR1 blockade on central noradrenergic neurotransmission. Our results suggest that some, but not all, noradrenergic neurons of the LC express FGFR1. On the LC noradrenergic neurons expressing FGFR1, PD173074 putatively had a robust inhibitory effect completely suppressing their firing activity; the LC noradrenergic neurons not expressing FGFR1 were putatively not affected by PD173074. Since the FGFR1 blockade putatively leads to the suppression of the LC noradrenergic neurons, the stimulatory effect of the FGF2 on these neurons is likely to be mediated via the FGFR1, similarly to the stimulatory effect of FGF2 on dopaminergic neurons, as found in our previous study (Grinchii et al., 2022).

With respect to 5-HT neurons of the DRN, we found that FGF2 decreased the frequency of their burst firing and percentage of the spikes occurring within the bursts. This effect was observed in male, but not in female rats. Since the burst firing of 5-HT neurons is more efficient in terms of neurotransmitter release from the nerve terminals (Cooper, 2002), FGF2 may inhibit central 5-HT neurotransmission. To our best knowledge, we were first to examine the effect of FGF2 on the excitability of the brain 5-HT neurons. It was however previously reported that FGF2 is present in the brain 5-HT neurons, suggesting an involvement in their development and/or functioning (Chadi et al., 1993). It was also reported that the therapeutic effect of the 5-HT transporter (SERT) inhibition by fluoxetine requires FGF2 (Simard et al., 2018). It is possible that the stimulatory effect of FGF2 on noradrenergic neurons, observed in our study, is also explained by the interaction between FGF2 and NAT. Future studies should be performed to examine this hypothesis.

We found that the selective blockade of the FGFR1 or FGFR2 receptors by the systemic PD173074 or i. c.v. lirafugratinib did not alter the excitability of 5-HT neurons of the DRN. Thus, the effect of FGF2 on 5-HT neurons of the DRN is not likely to be linked with FGF1 or FGFR2 receptors. With respect to the FGFR3, this receptor is primarily interacting with FGF8 (Chellaiah et al., 1999) and FGF9 (Santos-Ocampo et al., 1996) and its role in FGF2 signaling appears to be neglectable.

We found that the i. c.v. administration of the inhibitor of FGFR4 receptors, BLU9931, increased the percentage of spikes occurring within the bursts. It is thus possible that the inhibitory effect of FGF is mediated, at least in part, via FGFR4.

It was found that the mean firing rate of noradrenergic and 5-HT neurons, as well as the burst firing frequency of noradrenergic and the mean density of the spontaneously active 5-HT neurons, were higher in the males comparing to the females. Similar sex-related differences in the firing activity of 5-HT neurons were observed in the previous studies from our (Paliokha et al., 2026; Grinchii et al., 2024) and other laboratories (Robichaud and Debonnel, 2005). Interestingly, the effect of FGF2 on the excitability of noradrenergic and 5-HT neurons was detected in males, but not in females. Several mechanisms may explain why the effects of FGF2 on LC noradrenergic and DRN 5-HT neurons were more prominent in male rats. First, we observed baseline sex differences in the excitability of both neuronal populations, suggesting that the functional state of monoaminergic neurons differs between males and females even under control conditions. Such baseline differences may influence the magnitude and detectability of FGF2-induced changes in burst-related parameters. Second, gonadal hormones may modulate either FGFR expression or the intracellular coupling of FGFRs to downstream signaling pathways, including PI3K/AKT, ERK, JAK/STAT, PLCγ/Ca2+, and protein kinase C pathways. Therefore, the same FGF2 signal may produce different electrophysiological consequences depending on the hormonal and intracellular signaling context. Third, sex-dependent differences in autoreceptor tone, local inhibitory/excitatory inputs, or monoamine transporter function may indirectly shape the responsiveness of LC and DRN neurons to FGF2. Since the present study did not directly assess circulating sex hormones, estrous cycle phase, FGFR expression, or downstream signaling activation, these explanations remain hypothetical. Future studies combining electrophysiology with receptor expression analysis and hormonal status assessment will be required to determine the mechanisms underlying the sex-dependent sensitivity to FGF2. With respect to nigrostriatal and mesocorticolimbic dopaminergic neurons, similar effect of FGF2 and PD173074 were observed (Grinchii et al., 2023).

The opposite effects of FGF2 on LC noradrenergic and DRN 5-HT neurons may be relevant for understanding how FGF2 influences behaviors associated with substance use disorder and other neuropsychiatric conditions. Increased burst-related activity of LC noradrenergic neurons may enhance noradrenergic output to forebrain regions involved in arousal, stress responsiveness, attention, salience attribution, and cue reactivity. In the context of addiction, such an effect could facilitate the behavioral impact of drug- or alcohol-associated cues and stress-related triggers, both of which are important factors contributing to craving and relapse vulnerability. In contrast, reduced burst-related activity of DRN 5-HT neurons may decrease serotonergic modulation of circuits involved in impulse control, affective regulation, behavioral inhibition, and aversive processing. Thus, a combination of enhanced noradrenergic excitability and reduced serotonergic burst output could shift the balance of monoaminergic regulation toward increased arousal, cue responsiveness, and reward-seeking behavior, while weakening serotonergic restraint over impulsive or compulsive behavioral patterns. This interpretation is consistent with the previously reported pro-alcohol consumption effects of FGF2, although the present study did not directly assess behavior. Therefore, the behavioral relevance of the LC-DRN electrophysiological dissociation should be tested in future studies combining FGF2/FGFR manipulations with alcohol self-administration, stress-induced reinstatement, anxiety-like behavior, and depression-related behavioral paradigms.

Because neurons were not recorded longitudinally before and after drug administration, the present design does not allow classification of individual neurons as modulated or non-modulated by FGF2. Therefore, the conclusions are based on group-level electrophysiological differences rather than within-cell modulation.

All recordings were performed under chloral hydrate anesthesia. Chloral hydrate can influence neuronal excitability, synaptic transmission, autonomic tone, and monoaminergic neuronal activity. Because all experimental groups were recorded under the same anesthetic conditions, the group comparisons remain internally comparable. However, the present findings should be interpreted as effects observed under chloral hydrate anesthesia and may not fully reflect LC and DRN activity in awake animals. Future studies in awake or minimally restrained animals would be required to determine whether similar effects occur under physiological behavioral conditions.

Although the present recordings were performed using stereotaxic coordinates and electrophysiological criteria previously validated in our laboratory by histological verification, histological verification was not repeated in the present set of experiments. This should be considered a methodological limitation.

Concluding, the outcomes of the present study, together with the results of our previously published work (Grinchii et al., 2023), indicates that psychoactive effects of the FGF2 might be mediated, at least in part, via its interaction with the central monoaminergic circuits. The FGF2-catecholaminergic interactions are putatively mediated via FGFR1, and FGF2-5-HT-crosstalk-via FGFR4. These two receptors can be thus potential targets for the future CNS drugs.

## Data Availability

The data analyzed in this study is subject to the following licenses/restrictions: The data will be available on request. Requests to access these datasets should be directed to ED, eliyahu.dremencov@savba.sk.
